# Curcumin and Vitamin E Protect against Adverse Effects of Benzo[a]pyrene in Lung Epithelial Cells

**DOI:** 10.1371/journal.pone.0092992

**Published:** 2014-03-24

**Authors:** Wenbin Zhu, Meghan M. Cromie, Qingsong Cai, Tangfeng Lv, Kamaleshwar Singh, Weimin Gao

**Affiliations:** Department of Environmental Toxicology, The Institute of Environmental and Human Health, Texas Tech University, Lubbock, Texas, United States of America; Georgia Regents University, United States of America

## Abstract

Benzo[a]pyrene (BaP), a well-known environmental carcinogen, promotes oxidative stress and DNA damage. Curcumin and vitamin E (VE) have potent antioxidative activity that protects cells from oxidative stress and cellular damage. The objectives of the present study were to investigate the adverse effects of BaP on normal human lung epithelial cells (BEAS-2B), the potential protective effects of curcumin and VE against BaP-induced cellular damage, and the molecular mechanisms of action. MTT assay, flow cytometry, fluorescence microplate assay, HPLC, qRT-PCR, and western blot were performed to analyze cytotoxicity, cell cycle, reactive oxygen species (ROS), BaP diol-epoxidation (BPDE)-DNA adducts, gene expression, and protein expression, respectively. Curcumin or VE prevented cells from BaP-induced cell cycle arrest and growth inhibition, significantly suppressed BaP-induced ROS levels, and decreased BPDE-DNA adducts. While CYP1A1 and 1B1 were induced by BaP, these inductions were not significantly reduced by curcumin or VE. Moreover, the level of activated p53 and PARP-1 were significantly induced by BaP, whereas this induction was markedly reduced after curcumin and VE co-treatment. Survivin was significantly down-regulated by BaP, and curcumin significantly restored survivin expression in BaP-exposed cells. The ratio of Bax/Bcl-2 was also significantly increased in cells exposed to BaP and this increase was reversed by VE co-treatment. Taken together, BaP-induced cytotoxicity occurs through DNA damage, cell cycle arrest, ROS production, modulation of metabolizing enzymes, and the expression/activation of p53, PARP-1, survivin, and Bax/Bcl-2. Curcumin and VE could reverse some of these BaP-mediated alterations and therefore be effective natural compounds against the adverse effects of BaP in lung cells.

## Introduction

Lung cancer is the leading cause of cancer death worldwide and is estimated to be responsible for 1.38 million deaths in 2013 [1]. Cigarette smoking is the prominent cause of lung cancer, and polycyclic aromatic hydrocarbons (PAHs), such as benzo[a]pyrene (BaP), are the major carcinogens in cigarette smoke and play a critical role in lung carcinogenesis [2]. BaP is known to be the most potent carcinogen and has been listed as a *Group 1* carcinogen by the International Agency for Research on Cancer (*IARC)*. BaP can be metabolized to its ultimate carcinogen, BaP-7,8-diol-9,10-epoxide (BPDE), by cytochrome P450 (CYPs) enzymes. BPDE then can either be detoxified by Phase II enzymes or covalently bound to DNA and cause DNA damage, which can further lead to genetic mutation [3]. Research indicates that BaP elicits other toxic effects in target cells, such as oxidative stress. For instance, BaP metabolites involved in the redox cycle produce reactive oxygen species (ROS) including superoxide anion radicals (O_2_^-^) and hydrogen peroxide (H_2_O_2_) [4]. Genetic mutations and free radicals/ROS resulting from BaP exposure play a vital role in lung carcinogenesis [5].

The formation and persistence of BaP-DNA adducts is understood to be one of the first events in tumor initiation [6]. BPDE-DNA adducts play an essential role in BaP-induced DNA damage, and the quantity of adducts may be an important determinant of the cell destination in response to BaP exposure [7]. Besides the DNA-adducts, the induction of oxidative stress is another molecular mechanism through which BaP induces cytotoxicity. Excessive ROS has shown carcinogenic, teratogenic, and mutagenic potential in cells. For example, ROS can cause DNA damage resulting in the formation of 7,8-dihydro-8-oxo-2′-deoxyguanosine (8-oxo-dGuo) lesions [8], [9]. In response to DNA damage, the activation of cell cycle checkpoints is further needed to monitor the progress of the cell cycle. G1/S phase checkpoints prevent the replication of damaged DNA, while G2/M checkpoints inhibit the segregation of damaged chromosomes during mitosis [10]. Beyond oxidative and/or DNA damage, as well as alterations in the cell cycle, cells could respond to BaP through other molecular mechanisms.

The Phase I enzymes, CYP 1A1 and 1B1, and Phase II enzymes, superoxide dismutase (SOD) and catechol-O-methyl transferases (COMT) are some of the enzymes responsible in metabolism of BaP. Upon BaP exposure, Phase I enzymes are responsible for the transformation of BaP into its ultimate carcinogen, BPDE, while the Phase II drug-metabolizing enzyme, COMT, has been reported to inactivate BaP into less toxic or inactive metabolites [11]. Similarly, molecular targets such as p53, poly[ADP-ribose] polymerase 1 (PARP-1), and survivin could be activated by oxidative stress and/or genotoxic stress [12]-[14]. The activation of p53 by PAHs alleviates MDM2-dependent inhibition of p53 activity [15] and further controls the cell cycle and regulated apoptosis in mammalian cells [16], [17]. PARP-1 is a DNA damage signaling molecule implicated in several crucial cellular processes: DNA replication, transcription, DNA repair, apoptosis, and genome stability [18]. Survivin is a member of the inhibitor of apoptosis protein family and is another well-known protein critical to cell survival and it could possibly provide protection against cellular stress [19]. Finally, Bcl-2 is involved in cell survival promotion whereas Bax expresses pro-apoptotic properties [20], [21].

Numerous natural compounds have demonstrated anti-cancer and/or chemopreventive effects upon ingestion [22]. Curcumin, the active compound derived from turmeric, has exhibited chemopreventive potential against a variety of cancers, including lung cancer [23]–[25]. Many beneficial effects caused by curcumin are attributed to its antioxidative properties since it is a powerful scavenger of the superoxide anion, the hydroxyl radical, and nitrogen dioxide [26]–[28]. α-tocopherol, the most abundant and active vitamin E (VE) family member, also functions as a chain-breaking antioxidant that prevents the propagation of free radicals [29]. As a potent antioxidant, VE has been implicated in the prevention of several cancers, particularly lung cancer [30].

Although curcumin and VE can quench ROS formed from carcinogen exposure and may act as chemopreventive compounds functioning through their antioxidative properties, their molecular mechanisms against acute BaP exposure have not been fully characterized. The overall objective of this study was therefore to elucidate the role of curcumin and VE in cellular remediation after acute exposure to BaP. In the present study, normal human lung epithelial cells, BEAS-2B, were treated with BaP for 24 h, and then in an effort to prevent or eliminate cytotoxicity, oxidative stress, and DNA damage, cells were treated with curcumin or VE. Additionally, the molecular mechanisms, including the regulation of metabolizing enzymes, alterations of cell cycle, and modulation of p53/PARP-1/survivin/Bax/Bcl-2 were investigated.

## Methods and Materials

### Chemicals

Curcumin (98% pure), BaP, α-tocopherol succinate, and dimethylsulfoxide (DMSO) were purchased from Sigma Chemical Co. (MO, USA). Curcumin and α-tocopherol were dissolved in DMSO at a concentration of 50 μM and all the solutions were stored in a dark-colored bottle at −80°C as a stock solution. The stock was diluted to the required concentration with growth media immediately before use. 3-(4,5-Dimethylthiazol-2-yl)-2,5- diphenyltetrazolium bromide (MTT) was purchased from Usb Corporation (CA, USA). DNeasy Blood and Tissue kits were purchased from QIAGEN (MD, USA). Acetonitrile (ACN) and ammonium formate (AF) were purchased from Merck (Darmstadt, Germany) and HCl (1 N) was from Fisher Scientific (Fair Lawn, NJ). BaP-tetrol standards, including r-7,c-10,t-8,t-9-tetrahydroxy-7,8,9,10-tetrahydrobenzo[a]pyrene (BaP tetrol I-1), and r-7,t-9,t-10,t-8-tetrahydroxy-7,8,9,10-tetrahydrobenzo[a]pyrene (BaP tetrol I-2) were purchased from the MRIGlobal Chemical Carcinogen Reference Standard Repository (MO, USA).

### Cell culture

Human bronchial epithelial cell line, BEAS-2B, was obtained from American Type Culture Collection (ATCC). The cells were cultured as described in our previous study [31]. In brief, after the flasks/dishes/plates were coated, BEAS-2B cells were cultured in LHC-9 (Life Technology, MI) completed growth medium containing 50U penicillin/mL and 50 μg/mL streptomycin. The cells were then incubated at 37°C in 95% air and 5% CO_2_. At 80% confluence, cells were harvested using 0.05% trypsin/EDTA solution (HyClone) and were then sub-cultured.

### MTT assay

BEAS-2B cells were seeded into 96-well plates at a density of 1.3×10^4^ cells/well. Cells were treated with the following: 1) BaP alone (0–80 μM) for 24 and 48 h; 2) curcumin (0–80 μM) or VE (0–80 μM) individually for 24, 48, and 72 h; 3) co-treatment with BaP (5 μM) and curcumin (0–80 μM) for 24 h; and 4) co-treatment with BaP (5 μM) and VE (0–80 μM) for 24 h. The MTT solution (5 mg/mL) was added to the cells and incubated for 3 h at 37°C. After removal of the culture medium, the cells were dissolved by DMSO and absorbance was measured at 570 nm with a reference wavelength at 630 nm. The results were expressed as the percent of viable cells compared to the vehicle controls.

### ROS production

ROS formation in cells was detected using 2,7-dichlorofluorescein diacetate (DCFDA) (Sigma Chemical Co., MO) as described previously [32]. Briefly, cells were seeded into 96-well plates with different drug treatments for 24 h and were washed with 200 μL 1× phosphate buffered saline (PBS). After washing, the cells were incubated at 37°C in 100 μL 1× PBS containing 20 μM (final concentration) DCFDA for 45 min. Then, the fluorescence released was measured at an excitation of 485 nm and emission of 535 nm. The results were expressed as fold increase in fluorescence emission compared to the vehicle controls.

### Flow cytometry

For cell cycle analysis, a total of 2×10^5^ cells from each treatment group were collected and fixed in 70% ethanol for more than 24 h at 4°C. Cells were stained with Guava Cell Cycle Reagent (Millipore Corporation, MA) and run on a Guava EasyCyte Flow Cytometer (Millipore). A total of 5×10^3^ events were counted and the percentage of cells in the pre-G1, G0/G1, S, and G2/M phases of the cell cycle were determined using GuavaSoft software (Millipore). Each sample was run in triplicate.

### DNA adduct analysis

DNA adducts were measured for each DNA sample using high-performance liquid chromatography (HPLC) [33], [34]. Cells (2.5×10^6^ cells) were exposed to various test compounds for 24 h. After trypsinization, DNA was extracted from the cells using the DNeasy Blood and Tissue kit (QIAGEN Sciences, MD), according to the manufacturer's instructions, and the DNA quantity was estimated by Nano-Drop 1000 Spectrophotometer (Thermo Scientific, Waltham, MA) at 260/280 nm. For analysis, DNA samples were dissolved in 0.1 N HCl and acid hydrolyzed at 90°C for 3 h.

The BPDE tetrol adducts were determined by HPLC, model 1100 (Agilent Technologies, Wilmington, DE), equipped with a G1321A fluorescence detector, a G1313A Automatic Liquid Sampler, and a G1311A Quat Pump. ChemStation (Agilent Technologies) was used for instrument control, data acquisition, and analyses. In brief, 60 μL of each sample was injected onto a C18 column (5 μm, 4.6×250 mm; W. R. Grace & Co, OR). The column was eluted for 32 min at a flow rate of 1 mL/min. The mobile phase consisted of Solvent A (5% ACN with 10 mM AF) and Solvent B (100% ACN with 10 mM AF), and the gradient elution started with 10% Solution B for 5 min, ramped to 100% Solution B for 20 min, holding for 5 min, and returning to the initial gradient of 90∶10 (A∶B) and holding for 7 min. The excitation and emission wavelengths for the detector were 344 and 398 nm, respectively. Identification of the metabolites was accomplished by comparison of retention times of the samples, with the standards. The amount of adducts per μg of DNA sample were estimated using the standard curve generated by the fluorescence peak heights of an authentic BaP-tetrol standard. Relative amounts of BPDE tetrols in each group were compared to the BaP group, in which the adduct amount was set at 100%.

### Quantitative real-time PCR (qRT-PCR)

A one-step real time PCR (RT-PCR) kit with SYBR green was used for amplification of total RNA (50 ng). Reactions were set-up following the manufacturer's instructions (Bio-Rad, CA). Single-step RT-PCR amplifications were performed using the iCycler IQ (Bio-Rad) programmed for reverse transcription at 50°C for 15 min, denaturation and reverse transcriptase enzyme inactivation at 95°C for 5 min, followed by 40 cycles each containing 10 seconds for denaturation at 95°C and 30 seconds for annealing and extension at 60°C. Specificity of the PCR products was confirmed by melt curve analysis. Data were normalized to Ct values of GAPDH from the same sample and the fold-changes in the expression of each gene were calculated using the ΔΔCt method. A non-template control was included in each experiment. Primer sequences used are provided in Table 1.

**Table 1:** Forward and reverse primers of genes used in qRT-PCR.

Primer	Forward primer (5′to 3′)	Reverse primer (5′ to 3′)
GAPDH	GGTGGTCTCCTCTGACTTCAACA	GTTGCTGTAGCCAAATTCGTTGT
CYP1A1	TGGTCTCCCTTCTCTACACTCTTGT	ATTTTCCCTATTACATTAAATCAATGGTTCT
CYP1B1	CACTGCCAACACCTCTGTCTT	CAAGGAGCTCCATGGACTCT
CYP1A2	AACAAGGGACACAACGCTGAAT	GGAAGAGAAACAAGGGCTGAGT
CYP3A4	CTTCATCCAATGGACTGCATAAAT	TCCCAAGTATAACACTCTACACAGACAA
Bax	TTTGCTTCAGGGTTTCATCC	CAGTTGAAGTTGCCGTCAGA
Bcl-2	GGATGCCTTTGTGGAACTGT	AGCCTGCAGCTTTGTTTCAT

### Western blot analysis

After drug treatment for 24 h, the cells were washed and lysed in RIPA lysis buffer. Protein concentrations were measured using the Bio-Rad Bradford protein assay (Bio-Rad Life Science, Hercules, CA). Protein lysate was separated using 12% SDS-polyacrylamide gel electrophoresis and then transferred to polyvinylidene fluoride (PVDF) membranes. The immobilized proteins were then incubated in blocking buffer containing 3% nonfat, dry milk in 1×PBS and 0.1% Tween 20 (1× PBST). After blocking, the membranes were probed with the appropriate primary antibody overnight at 4°C. Immunoblotting was performed using phospho-p53 (Ser15) (p-p53), p53, PARP-1, COMT, SOD-1, and survivin (Santa Cruz Biotechnology, Santa Cruz, CA), and α-tubulin (for the internal control, Cell Signaling). After washing, the membranes were incubated with the corresponding secondary antibody (Santa Cruz) for 1 h at room temperature. After a brief incubation with enhanced chemiluminescence, the blots on the membrane were exposed to X-ray film (Fujifilm Corporation, Tokyo).

### Statistical analyses

Factorial analysis of variance (ANOVA) was performed to test the effects of BaP, curcumin, and/or VE on cell viability, ROS production, and DNA adducts. Probit analysis was performed to calculate the IC50s. Protein/band intensity in western blots was determined using Quantity One software and each protein tested was compared to the internal control, α-tubulin. One-way ANOVA was used to determine the difference in gene and protein expression between groups. All analyses were performed using SPSS 13.0 software. Differences at P<0.05 were considered statistically significant.

## Results

### Curcumin and VE decrease BaP-induced cytotoxicity

**Figure 1:**
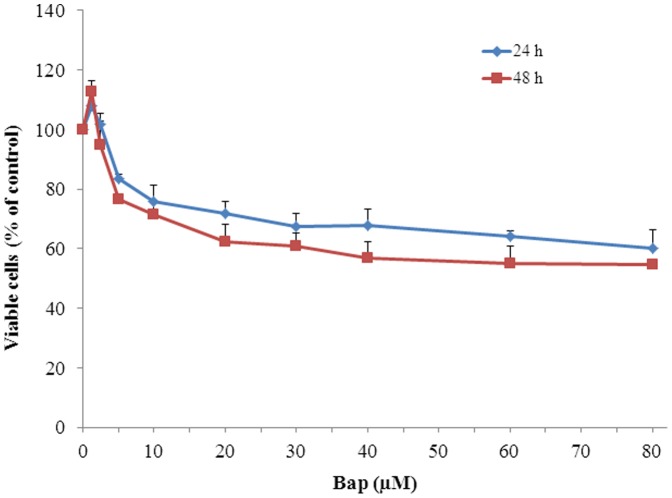
The cytotoxicity of BaP on BEAS-2B cells. The effect of BaP on cytotoxicity of human lung epithelial cell, BEAS-2B, was determined by MTT assay. After cells were seeded for 24 h, they were treated with 0-80 μM of BaP for 24 and 48 h. Data are expressed as the percentage of the value as compared to the cells treated with vehicle control (0.1% DMSO). Data represent as means ± SD, n = 6. Experiments performed in triplicate yielded similar results.

The effect of BaP on the cytotoxicity of BEAS-2B cells was determined by MTT assay. After 24 and 48 h of treatment, BaP (1.25–80 μM) was found to induce cytotoxicity in a dose-dependent manner (Figure 1). Since BaP at concentrations greater than 5 μM induced significant cytotoxicity in BEAS-2B cells compared to the control and 5 μM of BaP was an environmentally and occupationally relevant concentration, this dose was used in subsequent experiments.

**Figure 2:**
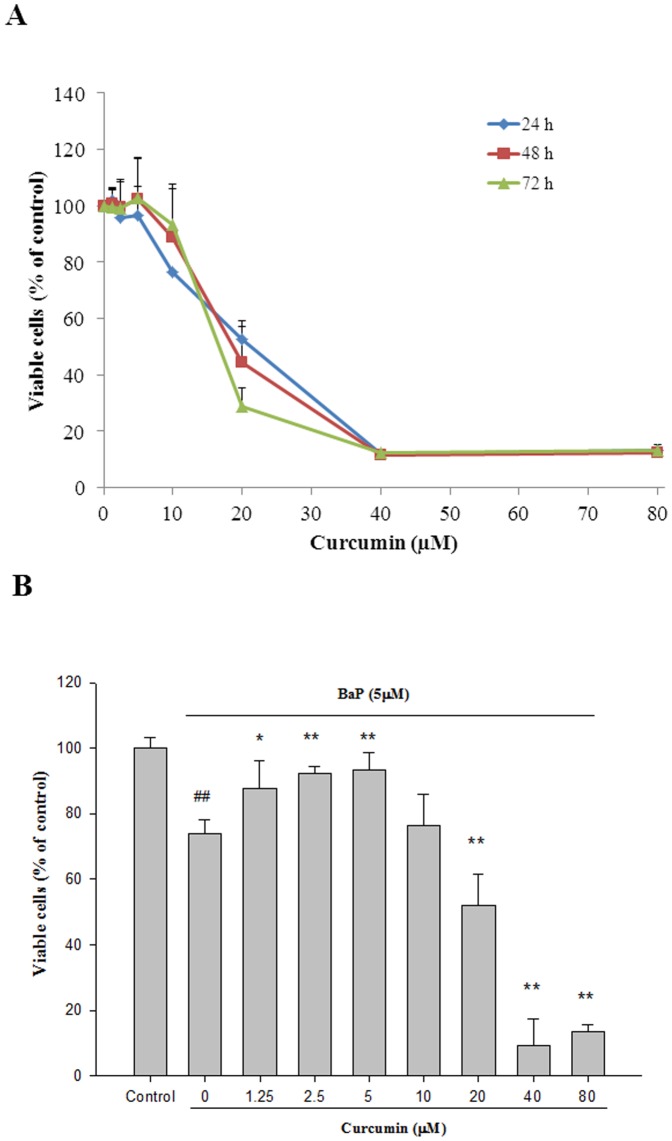
Cytoprotective effects of curcumin on BEAS-2B cells. (**A**) The cytotoxicity of curcumin. BEAS-2B cells were exposed to different concentrations of curcumin for 24, 48, and 72 h, and cytotoxicity was measured by MTT assay. Data are expressed as mean ± SD. n = 6. (**B**) Protective effect of curcumin against BaP-induced cytotoxicity. Co-treated with curcumin (0–80 μM) and 5 μM BaP for 24 h and cytotoxicity was measured by MTT assay. Values are means ± SD, n = 6. ## P<0.01, BaP alone compared to control; * P<0.05, ** P<0.01, BaP+Curcumin compared to BaP alone.

**Figure 3:**
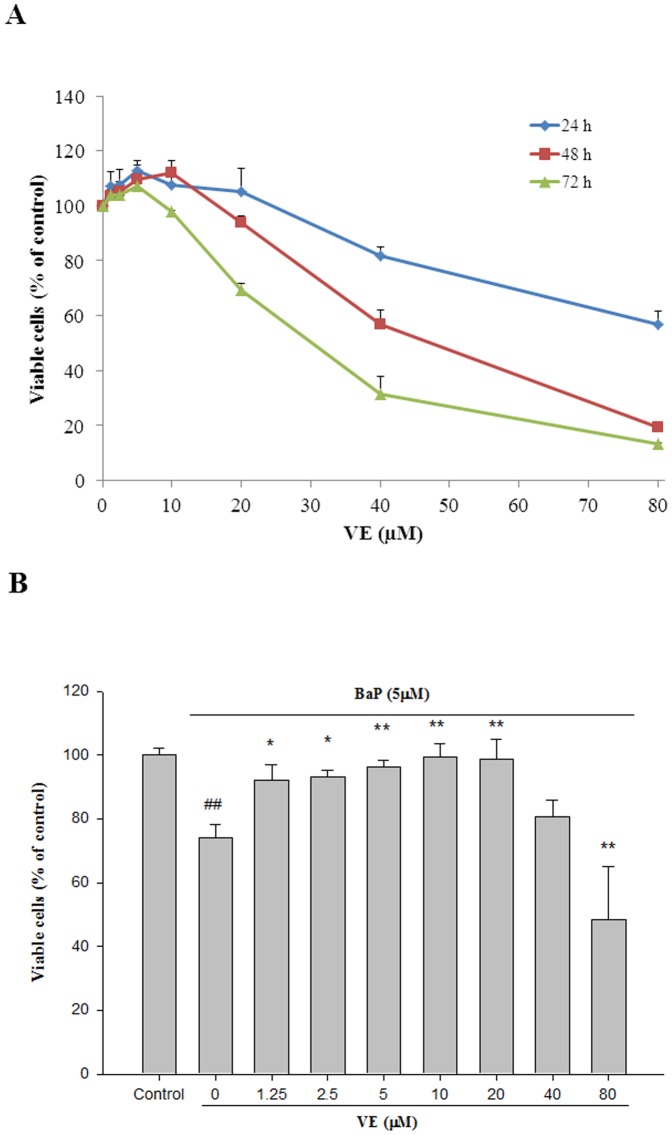
Cytoprotective effects of VE on BEAS-2B cells. (**A**) The cytotoxicity of VE. BEAS-2B cells were exposed to different concentrations of VE for 24, 48, and 72 h and then cytotoxicity was measured by MTT assay. Data are expressed as mean ± SD. n = 6. (**B**) Protective effect of VE against BaP-induced cytotoxicity. Co-treated with VE (0–80 μM) and 5 μM BaP for 24 h and cytotoxicity was measured by MTT assay. Values are means ± SD, n = 6. ## P<0.01, BaP alone compared to control; * P<0.05, ** P<0.01, BaP+VE compared to BaP alone.

To determine curcumin- or VE- induced cytotoxicity in BEAS-2B cells, cells were cultured in the absence (vehicle control) or presence of curcumin or VE at concentrations of 1.25–80 μM. As shown in Figures 2A & 3A, BEAS-2B cell growth was significantly inhibited in a dose- and time-dependent manner (P<0.01). The IC50s of curcumin in BEAS-2B cells are 21, 19, and 17 μM at 24, 48, and 72 h, respectively. For VE, IC50s can be observed at 48 and 72 h at 47 and 35 μM, respectively.

The possible protective effects of curcumin and VE on BaP-induced cytotoxicity were then examined. As shown in Figure 2B, the number of viable cells exposed to 5 μM BaP was 76% of the control, and the number of viable cells co-treated with curcumin at 1.25, 2.5, and 5 μM increased in a significant manner to 88, 92, and 93%, respectively (P<0.05, BaP+Curcumin *vs.* BaP). Similar results can be found in Figure 3B, when cells co-treated with 5, 10, and 20 μM VE viable cells increased y to 96, 99, and 99%, respectively (P<0.01, BaP+VE *vs.* BaP). Based on the cytoprotective data, 5 μM of curcumin and 20 μM of VE were used in the subsequent experiments.

### Curcumin and VE act as a ROS scavenger

**Figure 4:**
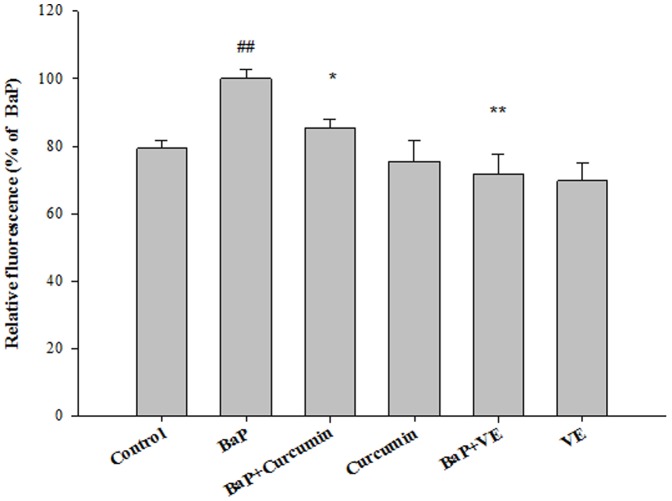
The effects of antioxidants on BaP-induced ROS production. Data are expressed as means ± SD of three replicate experiments. ## P<0.01, BaP alone compared to control; * P<0.05, ** P<0.01, co-treated group compared to BaP alone.

As shown in Figure 4, treatment of cells with 5 μM BaP induced a 20% increase in ROS production compared to the control (P<0.05). Treatment with curcumin or VE alone did not alter ROS levels compared to the untreated control group. However, BaP-induced ROS production was significantly reduced to 85% by curcumin (P<0.05) and to 72% by VE (P<0.01).

### Effects of curcumin and VE on BaP-induced DNA adducts

**Figure 5:**
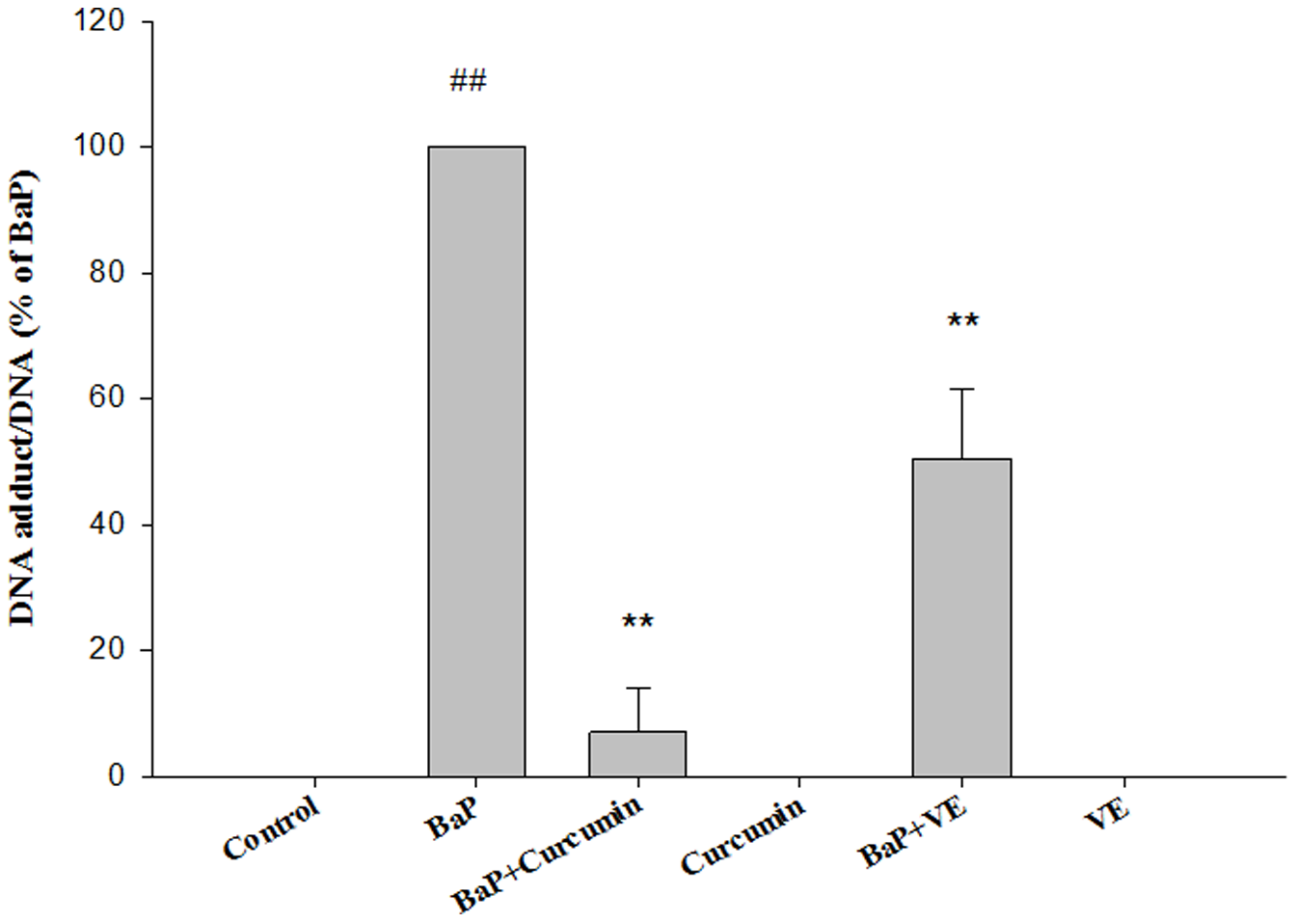
Effects of antioxidants on BaP-induced DNA adducts. The total DNA was extracted and adduct levels were quantified by HPLC as described in materials and methods. Data shown are means ± SD of three replicate experiments. ## P<0.01, BaP alone compared to control; ** P<0.01, co-treated group compared to BaP alone.

The effects of curcumin and VE on BaP-induced DNA adducts are presented in Figure 5. The relative DNA adduct amounts show that BaP alone formed significant levels of DNA adducts after the exposure of BEAS-2B cells to 5 μM BaP for 24 h (P<0.01). Co-treatment of BaP with curcumin or VE resulted in the prevention of BPDE-DNA adduct formation in cells. Compared to BaP alone, the relative BPDE-DNA adducts of cells co-treated with 5 μM curcumin or 20 μM VE significantly decreased to 7% or 50%, respectively (P<0.01, BaP+Curcumin *vs.* BaP; P<0.01, BaP + VE *vs.* BaP).

### Effects of antioxidants on BaP-induced cell cycle phase distribution

Flow cytometry analysis revealed that treatment with 5 μM BaP for 24 h significantly decreased the number of cells in the G0/G1 phase by 11.9% (P<0.05), with a concomitant increase in the G2/M phase from 23.2% to 32.7% (Table 2). The G2/M phase accumulation induced by BaP was suppressed 3.2% by curcumin and 4.0% by VE. In addition, flow cytometry analysis showed no apoptotic cells in any groups (data not shown).

**Table 2:** Effects of antioxidants on BaP-induced cell cycle of BEAS-2B cells.

	G0/G1	S	G2/M
Control	63.9±3.12	11.5±0.10	23.2±2.90
BAP	52.0±0.38##	13.9±0.68	32.7±0.25##
BAP+Curcumin	53.3±0.36*	15.1±0.43	29.5±0.64*
Curcumin	59.2±0.87	12.3±1.79	26.1±2.40
BAP+VE	54.2±3.11	15.5±1.67	28.7±1.55*
VE	59.9±1.14	10.6±1.37	27.6±2.11

## P<0.01 in comparison to control.

* P<0.05 in comparison to BaP alone.

### Effect of curcumin and VE on BaP-involved metabolism

**Figure 6:**
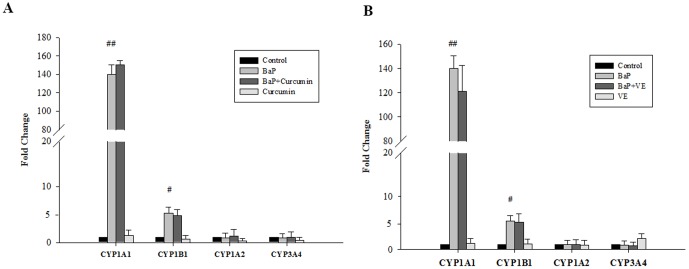
Effect of antioxidants on BaP-involved metabolism. (**A**) Quantitative RT-PCR analysis of CYP gene expressions after BEAS-2B cells were treated with BaP and/or curcumin. (**B**) Quantitative RT-PCR analysis of CYP gene expression after BEAS-2B cells were treated with BaP and/or VE. For quantitative RT-PCR analysis, total RNA isolated from BEAS-2B cells with 24 h treatment was used to perform a one-step quantitative RT-PCR as described in materials and methods. Cycle threshold value (Ct value) of each gene was normalized to the Ct value of housekeeping gene, GAPDH, obtained from the same sample. Data shown are means ± SD of three replicate experiments. # P<0.05, ## P<0.01, BaP alone compared to control. Data are means ± SD, n = 3. * P<0.05 compared to BaP alone.

The effects of antioxidants on the BaP-induced expression of the CYP1 family (CYP1A1, 1B1, and 1A2) and CYP3A4 in BEAS-2B cells were investigated. CYP1A1 and CYP1B1 mRNA expression levels in the 24 h BaP-treated group were 140-fold (P<0.01) and 6-fold (P<0.05) greater than the control group, respectively. However, BaP did not significantly induce CYP1A2 and CYP3A4 mRNA expression (Figures 6A & 6B). Furthermore, we explored whether the suppressive effect of antioxidants on BaP-DNA adduct formation was mediated through metabolic enzymes. Although curcumin and VE markedly suppressed BaP-DNA adduct formation, CYP gene expression was not significantly changed after co-treatment with 5 μM curcumin or 20 μM VE (Figures 6A & 6B).

The effects of BaP on Phase II enzymes, COMT and SOD-1, were evaluated by western blot (data not shown). BaP-treated cells did not show significant changes in SOD-1 protein expression compared to the vehicle control, while curcumin and VE did not alter SOD-1 expression. COMT western blots demonstrated that membrane-bound COMT had no significant changes among any groups but soluble COMT was significantly up-regulated in BaP-exposed cells after VE treatment compare to that in BaP alone (P<0.05; data not shown).

### Effects of curcumin and VE on protein expression and/or activation of p53, PARP-1, and survivin, and mRNA expression of Bax/Bcl-2

**Figure 7:**
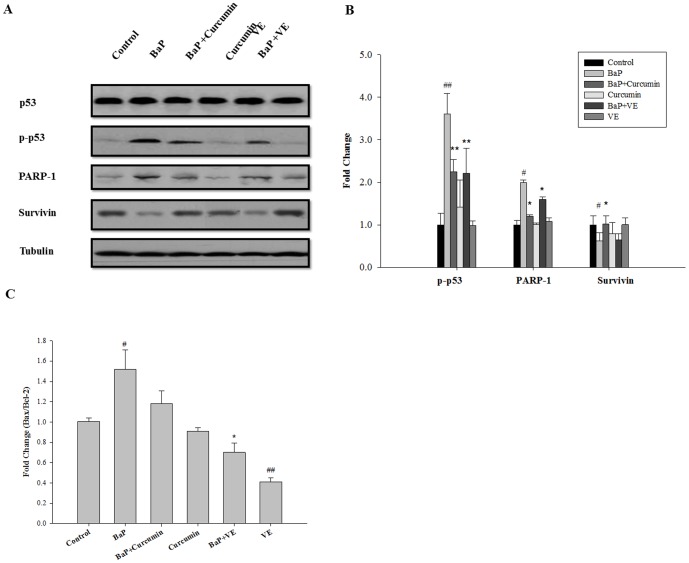
Effects of antioxidants on p53, p-p53, PARP-1, survivin, and Bax/Bcl-2. (**A**) Western blot analysis of protein expressions of total p53, p-p53 (Ser15), PARP-1, and survivin in BEAS-2B cells. The western blot was performed as described in materials and methods, and blots were also probed for α-tubulin to confirm equal protein loading. (**B**) The fold change of p-p53, PARP-1, and survivin in BEAS-2B cells treated with BaP, curcumin and/or VE compared to vehicle control. The intensity of each band was quantified using Quantity One software. (**C**) Quantitative RT-PCR analysis of Bax/Bcl-2 ratio after BEAS-2B cells were treated with BaP, curcumin and/or VE. Data are means ± SD, n = 3. # P<0.05, ## P<0.01, BaP alone compared to control; * P<0.05, ** P<0.01, co-treated group compared to BaP alone.

As shown in Figures 7A & 7B, further analysis of the p53 pathway showed no difference in the level of total cellular p53 between control- and BaP-treated cells. However, phosphorylation of p53 at Ser15 (p-p53) was markedly increased following BaP exposure in BEAS-2B cells (P<0.01). Although curcumin or VE by itself did not alter the expression level of p-p53 compared to the control, BaP-induced p-p53 was significantly diminished in the presence of 5 μM curcumin and 20 μM VE (P<0.01). After 24 h of BaP exposure, PARP-1 protein expression significantly increased compared to the vehicle control (P<0.05) and co-treatment with curcumin or VE resulted in a significant decrease in BaP-induced PARP-1 (P<0.05) (Figures 7A & 7B). Survivin displayed a significant decrease in protein expression after BaP exposure (P<0.05, Figures 7A & 7B). Curcumin and VE by itself did not alter the expression levels of survivin compared to the control. However, exposed cells treated with BaP+Curcumin significantly up-regulated survivin expression compared to the BaP group (P<0.05). Finally, the ratio of Bax/Bcl-2 was significantly increased in cells treated with BaP (P<0.05), but decreased in cells treated with VE compared to the vehicle control (P<0.01). Moreover, the ratio of Bax/Bcl-2 was significantly decreased after cells were treated with BaP+VE compared to those with BaP alone (P<0.05).

## Discussion

In the present study, the antioxidants curcumin and VE demonstrated significant cytoprotective effects against BaP-induced cytotoxicity in BEAS-2B cells by decreasing ROS production. Furthermore, increased p53 activation and PARP-1 expression induced by BaP were reduced after treatment with curcumin and VE. Down-regulation of survivin occurred after BaP exposure, but curcumin treatment resulted in a significant increase in survivin expression in cells exposed to BaP. To our knowledge, there is no report of the protective role of curcumin and VE against short-term BaP-induced cellular damage in human normal lung epithelial cells. Therefore, this is the first study demonstrating the protective potential of curcumin and VE and their mechanisms of action in normal lung epithelial cells after acute BaP exposure. Both curcumin and VE shared similar chemoprotective effects against BaP exposure potentially through their common antioxidative properties, as well as other unique pathways to these two natural compounds.

The dose of BaP used in this experiment, 5 μM, is regarded as an environmentally and occupationally relevant concentration [31], [35], [36], and this dose is a commonly used concentration in toxicity studies using human cell lines or tissues [35]–[38]. In the present study, cytotoxicity and DNA damage were observed in the BEAS-2B cells at this concentration, suggesting this dose could be potentially harmful for human populations that are exposed to BaP through environmental and/or occupational sources. However, no apoptotic cells were observed after this short-term BaP exposure, suggesting the occurrence of cellular growth inhibition/arrest instead of apoptosis after acute BaP exposure. Curcumin has been shown to exhibit pleiotropic effects on cell death and survival, such as at high concentrations it induces apoptosis or inhibits cell proliferation; however, at low concentrations, it protects cells from various toxins [39], [40]. In the present study, curcumin at a low, non-cytotoxic concentration of 5 μM, protected against BaP-induced cytotoxicity. This is consistent with other studies showing the protective effects of curcumin at this dose [41], [42]. Similar to curcumin, VE has been recognized to have biphasic attributes [43]. In the present study, curcumin (5 μM) and VE (20 μM) were selected because these natural compounds showed no effects on cytotoxicity whereas this dose had the greatest protective effects against BaP-induced cytotoxicity.

The protective effects of curcumin and/or VE against oxidative damage have been reported in both animal and *in vitro* studies [44]–[46]. In the present study, both curcumin and VE attenuated BaP-induced ROS production, demonstrating the direct scavenging effect of these antioxidants on free radicals. These results are consistent with other studies that confirm the protective effects of curcumin and VE against oxidative stress related to BaP metabolism [47], [48]. Given the adverse cytotoxic effects resulted from ROS, the reduction in BaP-induced ROS by curcumin or VE treatment could lead to decreased cytotoxicity in BaP treated BEAS-2B cells. Co-treatment with BaP and curcumin or VE resulted in the significant reduction of BPDE-DNA adduct formation in BEAS-2B cells. It has been reported that the formation of DNA adducts induced by dimethyl benzanthracene (carcinogenic PAH) were suppressed by curcumin [49]. Similarly, polyphenols inhibited BaP-derived DNA adduct formation *in vitro* in a dose-dependent manner [50]. Currently, there are no studies reporting the attenuation of DNA adduct formation in BaP-treated cells after VE exposure, and the present study is the first to report the protective effects of VE against DNA damage caused by short-term BaP exposure. The diminished DNA damage and ROS induction after curcumin and VE treatment was further reflected by the cell cycle analysis. BaP induced cell cycle arrest at the G2/M phase, which is consistent with previous findings [10]. Although 5 μM of curcumin and 20 μM of VE did not change the cell cycle of BEAS-2B with individual treatment, the fraction of cells in the G2/M phase after co-treatment of BaP and curcumin or VE were slightly but significantly decreased compared to the BaP alone group.

Among the four Phase I genes (CYP1A1/1B1/1A2/3A4) measured, the mRNA level of CYP1B1 was the most abundant in BEAS-2B cells. After BaP treatment, CYP1A1 and 1B1 were found to be significantly induced. These findings are consistent with previous studies suggesting that CYP1A1 and CYP1B1 expression in lung tissue from smokers and ex-smokers is significantly greater than in non-smokers [51], [52]. Additionally, the expression levels of the four CYP genes analyzed were not significantly modified after BaP-treated cells were co-treated with curcumin and VE. These data reveal that the decreased DNA damage after BaP exposed-cells co-treated with curcumin or VE is not a result of the induction/inhibition of CYP genes involved in BaP metabolism. The Phase II enzyme, SOD, is essential for the elimination of ROS and studies have observed that in rats, a correlation between increased SOD expression and BaP exposure exists [53] and similarly, COMT has been known to detoxify B[a]P-7,8-dione [54]. The unchanged SOD-1 and COMT protein levels after BaP exposure observed in the present study suggest that other Phase II enzymes might be involved in the detoxification of BaP-induced damage, which requires further study.

BaP-induced oxidative stress and mitochondrial dysfunction can induce p53 activation and aggravate p53-dependent apoptosis [15], [16]. In this study, it was found that co-treatment of BEAS-2B cells with curcumin or VE could reduce BaP-induced cytotoxicity and G2/M cell cycle arrest, as well as DNA adducts in BEAS-2B cells. These findings were further supported by the fact that BaP-induced p53 activation (phosphorylated at Ser15 of p53) and PARP-1 protein expression were attenuated after cells were co-treated with curcumin or VE. It is recognized that phosphorylation of Ser15 in p53 is an early critical event in the stabilization and activation of p53 in response to genotoxicity [55]. The decreased BaP-activated phospho-p53 expression could possibly be attributed to the decline in oxidative stress and DNA damage in BaP-exposed cells by both curcumin and VE. In line with our findings, Tao *et al* found that BaP significantly augmented PARP-1 expression in human bronchial epithelial cells [14]. Upon VE and curcumin treatment, BaP-induced PARP-1 levels were significantly decreased, implicating a reduction in DNA damage. In addition to p53 and PARP-1, survivin and Bax/Bcl-2 played critical roles in cell survival signaling [56], [57]. A significant decrease in survivin expression occurred in cells exposed to BaP, further validating the cytotoxicity data. Curcumin elicited a significant increase in survivin expression that could potentially result in the survival of the BaP-damaged cells. Furthermore, excess Bax can be deleterious to cell survival due to the promotion of Bax homodimers, while excess Bcl-2 was associated with cellular protection [20], [21]. The decreased ratio of Bax/Bcl-2 observed after VE treatment further indicated that the promoted cell growth/survival could be regulated by the Bcl-2 family in addition to the other signaling molecules such as p53, PARP-1, and survivin.

In summary, the exposure of BaP to normal human lung epithelial cells induced cytotoxicity, ROS generation, DNA damage, Phase I enzymes, cell cycle arrest, and p53/PARP-1/survivin alterations. Both curcumin and VE by itself showed no adverse effects but provided protection against BaP-induced cytotoxicity through reducing ROS and DNA damage, and/or altering p53/PARP-1/survivin/Bax/Bcl-2 expression/activation in lung BEAS-2B cells. Although this study revealed curcumin and VE to be promising chemoprotective agents, further studies are needed to address the effectiveness and molecular mechanisms underlying the protective effects of curcumin and VE in BaP-induced lung carcinogenesis using *in vitro* long-term exposure models as well as *in vivo* animal models.
